# Statistical Considerations for Subjective Visual Vertical and Subjective Visual Horizontal Assessment in Normal Subjects

**DOI:** 10.1097/ONO.0000000000000044

**Published:** 2023-12-13

**Authors:** Carey D. Balaban, Erin Williams, Cynthia L. Holland, Alexander Kiderman, Anthony P. Kontos, Michael E. Hoffer

**Affiliations:** 1 Departments of Otolaryngology, Neurobiology, Communication Sciences and Disorders, Bioengineering, and Mechanical Engineering and Materials Science, University of Pittsburgh, Pittsburgh, PA; 2 Departments of Otolaryngology; 3 Biomedical Engineering; 4 Department of Orthopaedic Surgery, University of Pittsburgh, Pittsburgh, PA and Concussion Research Laboratory, UPMC Sports Medicine Concussion Program, University of Pittsburgh Medical Center, Pittsburgh, PA; 5 Neurolign USA, Inc., Pittsburgh, PA; 6 Neurological Surgery; 7 Sports Performance and Wellness Institute, University of Miami, Miami, FL.

**Keywords:** Subjective visual vertical, Subjective visual horizontal, Normative values, Sex differences, Preset effect

## Abstract

**Objectives:**

Judgments of the subjective visual vertical (SVV) and subjective visual horizontal (SVH) while seated upright are commonly included in standard clinical test batteries for vestibular function. We examined SVV and SVH data from retrospective control to assess their statistical distributions and normative values for magnitudes of the preset effect, sex differences, and fixed-head versus head-free device platforms for assessment.

**Methods:**

Retrospective clinical SVV and SVH data from 2 test platforms, Neuro-otologic Test Center (NOTC) and the Neurolign Dx 100 (I-Portal Portable Assessment System Nystagmograph) were analyzed statistically (SPSS and MATLAB software) for 408 healthy male and female civilians and military service members, aged 18–50 years.

**Results:**

No prominent age-related effects were observed. The preset angle effects for both SVV and SVH, and their deviations from orthogonality, agree in magnitude with previous reports. Differences attributable to interactions with device type and sex are of small magnitude. Analyses confirmed that common clinical measure for SVV and SVH, the average of equal numbers of clockwise and counterclockwise preset trials, was not significantly affected by the test device or sex of the subject. Finally, distributional analyses failed to reject the hypothesis of underlying Gaussian distributions for the clinical metrics.

**Conclusions:**

*z* scores based on these normative findings can be used for objective detection of outliers from normal functional limits in the clinic.

Subjective visual vertical (SVV) and subjective visual horizontal (SVH) determinations are multisensory constructs, influenced by vestibular, visual, and haptic inputs (1). Aubert (2) reported that head and body tilt at relatively small angles altered the perceived orientation of an illuminated vertical line in a completely dark room. This bias in the direction of the tilt has been termed the Aubert effect, as compared to the Müller (or Entgegengesetzt effect) effect (3), which is a bias of the estimate in the opposite direction for larger angles of tilt. These biases in perception of the upright tilt were investigated extensively in perceptual experiments (4–11) and formed the basis for concepts of the roles of biological factors in perceptual field dependence (12–14). More recent studies (15,16) have presented convincing evidence that these perceptual effects represent an interaction between distinct head position-based gravity and haptic body-based gravity percepts. This information is important to inform the interpretation of clinically meaningful SVV and SVH findings in patients with vestibular and related disorders in comparison to those who are normal/healthy. However, the statistical impact of these perceptual effects on SVV and SVH test results in normal, healthy subjects has not been assessed.

The clinical use of SVV and SVH judgments from upright patients was introduced for neurologic and neurotologic diagnosis of peripheral vestibular and spatially circumscribed central neurological disorders by Friedmann (17), and it was later confirmed that the head-based estimates and body-based estimates of verticality are independent in normal subjects and patients with vestibular disorders (16). The reader is referred to the comprehensive review by Dieterich and Brandt (18) for their role in the assessment of balance disorders of central and peripheral origin. These estimates are now part of standard clinical tests of perceived verticality and horizontality, but the statistical properties of outcome measures from a large normative data set have not been summarized in the open literature. The current study analyzes data from a large sample of healthy subjects who performed SVV and SVH tasks using either a virtual heads-up display or a laser line projected in a darkened booth while seated upright in a clinical setting. For these tasks, the seated subjects are asked to adjust at light bar from a preset angle (clockwise [CW] or counterclockwise [CCW] rotation, once at 20 degrees and once at 25 degrees) to either a vertical (SVV) or horizontal (SVH) orientation using buttons that rotate the bar in 0.1-degree increments. This permitted estimation of the magnitudes and statistical distributions allowed us to evaluate several effects that have been described in the literature as sources of potential bias in clinical measurements. First, we examined the magnitude of the preset angle effect, described as a small bias of the estimates in the direction of the preset deviation of the linear marker (19). Second, we assessed the magnitude of the reported lack of orthogonality of SVV and SVH (20). Finally, we evaluated the magnitudes of sex differences in SVV and SVH judgments that have been reported previously (Luyat et al. 2012 (21)). We also explored age-related effects and the influence of testing devices on participant performance.

## METHODS

### Design and Participants

This study used deidentified cross-sectional data from 408 healthy subjects (159 females and 249 males) obtained by informed consent with IRB approval, in the course of previously published studies and ongoing studies at the University of Pittsburgh, University of Miami, and Naval Medical Center-San Diego. Exclusion criteria included:

1. A history of mild traumatic brain injury (mTBI) within the last 12 months or persistence of any prior mTBI-related symptoms at the time of enrollment.
2. A history of moderate to severe traumatic brain injury characterized by penetrating head trauma, Glasgow Coma Scale <13 at the time of injury, an associated loss of consciousness >30 minutes or amnesia >24 hours, or an associated subdural or epidural hemorrhage.
3. Prior disorders of hearing and balance including Meniere disease, multiple sclerosis, vestibular neuritis, vestibular schwannoma, and sudden sensorineural hearing loss.
4. A history of tumor of the brain or central nervous system.
5. A history of diagnosed psychiatric disorder (eg, depression, schizophrenia).
6. Documented neurological disorders (eg, Epilepsy, stroke, dementia, migraine).
7. Pregnancy (females by self-designation).
8. Presence of severe aphasia.

### Measures

Two testing systems were used to perform SVV and SVH tests, the Neuro-otologic Test Center (NOTC) and the Neurolign Dx 100 (I-Portal Portable Assessment System Nystagmograph [I-PAS]).

#### Neuro-otologic Test Center (NOTC)

The NOTC was used to test 258 subjects (81 females and 177 males). It is a Food and Drug Administration (FDA)-approved testing system, which is installed inside a circular, lightproof booth (model no. RCS-035) manufactured by Neurolign USA, LLC (formerly known as Neuro Kinetics, Inc., Pittsburgh, PA). Subjects are seated upright inside the booth 1 m from the black, smooth round enclosure surface. Subjects use 2 button controls installed on left and right chair handles to rotate SVV and SVH lines. SVV and SVH lines are created by 2-axis galvanometer motor and mirror assemblies that provide 2-dimensional scanning with 3-mm red laser. The moving-magnet galvanometers deliver line images at high speed, high resolution, and high repeatability (8 mrad), while integrated optical position detectors ensure precise control, high linearity, and low drift. Scan optical angle is 20 degrees projected at 1 m from subject on the round enclosure and is 35 cm long.

#### Neurolign Dx 100

The Neurolign Dx 100 (formerly known as I-PAS) was used to test 150 subjects (72 females and 78 males). It is an FDA-approved virtual reality (VR) goggle set with a built in eye-tracking device, manufactured by Neurolign USA, LLC (formerly known as Neuro Kinetics, Inc., Pittsburgh, PA). The Neurolign Dx 100 is a portable and compact head-mounted VR goggle set, equipped with high-speed digital infrared cameras (940 nm, sampling rate of 100 frames/S) that capture high-resolution images of eye movements in response to light stimuli. A hand-held apparatus with response buttons records participants’ manual input to SVV and SVH tests.

#### SVV and SVH Testing

SVV and SVH stimuli are presented in VR heads-up display with 60-degree field of view and resolution of 2560 × 1440 pixels. To eliminate subject’s ability to use display edges and pixels as a reference, a black background is used with circular visible area displaying SVV and SVH stimuli. SVV and SVH presentations are made from 4 dots with diffused edges. Subjects use handheld box with 2 buttons that rotate preset target lines in the CW (right button) and CCW (left button) directions to align with the perceived vertical or horizontal positions. Each subject repeats SVV and SVH tests for minimum 4 times, with randomly selected positive and negative preset angles of SVV and SVH (referenced to the display coordinate frame). The responses are recorded in degrees of rotation relative to either earth vertical (SVV) or earth horizontal (SVH), with the CW direction defined as positive.

### Statistical Analysis

Statistical analyses described below were performed with IBM SPSS and in MATLAB (MathWorks, Natick, MA).

## RESULTS

### Demographics

**FIG. 1.**
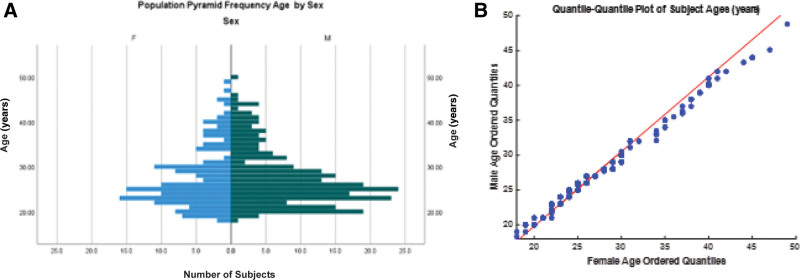
Age distribution. *A*, The age distributions of female (F) and male (M) subjects are shown in histograms. *B*, The quantile-quantile plot confirms the strong similarity of the distributions across the represented age range of the subject pool (unity relationship shown as a red line).

The ages of the female and male subjects were similarly distributed, as shown by the histograms (A) and the linear relationship in the sample quantile-quantile plot (B) in Figure 1. Kolmogorov-Smirnov tests rejected hypotheses of single normal, lognormal, and uniform distributions. Summary statistics demonstrated no difference between the ages of female (age mean: 27.6 years, SEM: 0.6 years, range: 18–49 years, age median: 25.5 years, age mode: 23.0 years, quartiles: 23.0, 30.0 years) and male subjects (age mean: 27.7 years, SEM: 0.4 years, range: 18–50 years, age median: 26.0 years, mode: 25.0 years, quartiles: 23.0, 30.25 years). Results of the Mann-Whitney test (*P* > 0.6) retained the hypothesis of equal distribution of females and males. A Kruskal-Wallis (independent samples) test showed no significant age differences between the male and female cohorts tested with either the NOTC or VR devices (statistic [adjusted for ties] =1.695, 3 degrees of freedom, *P* > 0.6).

### Analysis of SVV and SVH

Analysis of variance (ANOVA) was performed in IBM SPSS with 2 repeated measures factors, spatial estimate axis (SVV or SVH) and visual preset direction (CW or CCW rotation), and with 2 between-subject factors, sex (female or male) and device (chair [NOTC] or I-PAS/Dx-100 VR display). There was a strong, significant main effect for the visual preset direction (*F*_1392_ = 151.8, *P* < 0.001), confirming the preset effect in the literature. There were also 3 significant interactions effects. First, there was a strong visual preset direction × spatial estimate axis interaction (*F*_1392_ = 23.0, *P* < 0.001), indicating that preset effects differed for SVV and SVH. Second, there was a visual preset direction × spatial estimate axis × device interaction (*F*_1392_ = 4.3, *P* < 0.05), which suggested device-dependent effects. Finally, a prominent visual preset direction × spatial estimate axis × device × sex interaction (*F*_1392_ = 7.0, *P* < 0.01) indicated consistent differences between responses of females and males. Findings that produce these effects will be described in sequence.

**FIG. 2.**
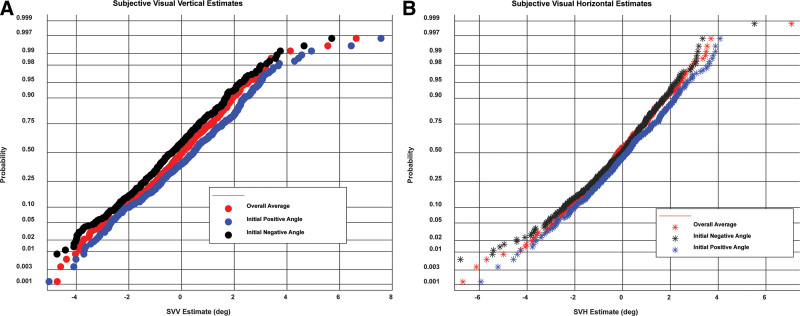
Preset effects for positive (CW) and negative (CCW) initial rotations of the vertical or horizontal line. The data (in degrees re: earth vertical [SVV] or earth horizontal [SVH]) were pooled across devices and sexes for these graphs. The cumulative distribution functions of the CW preset (initial positive angle) and CCW preset (initial negative angle) trials are shown in full normal probability plots for SVV (*A*) and SVH (*B*). The standard clinical metric, the average SVV or SVH across preset trial, is also shown. Note the linearity of the normal probability plots and the preset effect appears as a constant offset between data from CW and CCW preset trials. CW indicates clockwise; CCW, counterclockwise; SVV indicates subjective visual vertical; SVH, subjective visual horizontal.

**FIG. 3.**
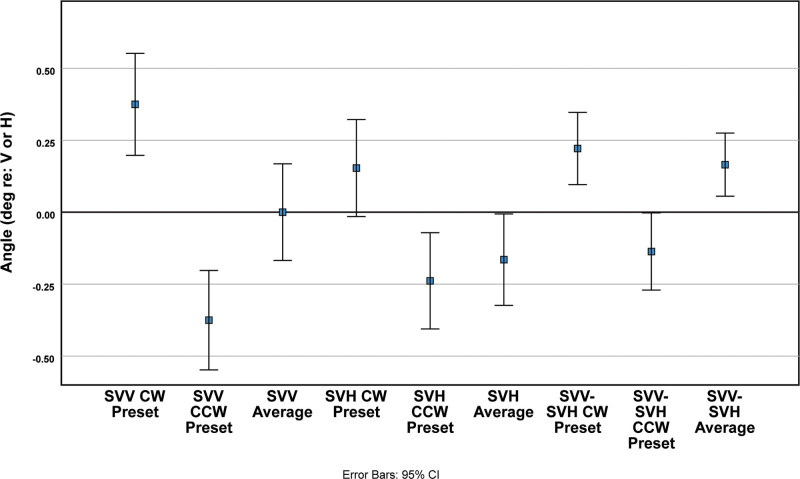
Preset effect in the pooled data (across devices and sexes) for subjective visual vertical (SVV) and subjective visual horizontal (SVH) estimates. The estimates are shown for positive (CW) and negative (CCW) preset values, as well as the average value SVV and SVH determinations and the difference between those estimates (SVV-SVH). See text for detailed description.

The main effect of visual preset direction and the visual preset direction × spatial estimate axis interaction effect indicate properties of the data pooled across sexes and test devices. Kolmogorov-Smirnov tests (Lilliefors correction) retained the null hypothesis of normality for the distributions of the subjects’ SVV for initial positive (CW) displacement, initial negative (CCW) displacement, and overall average; normal probability plots are linear (Fig. 2). These tests also retained the hypothesis of normality for the initial positive displacement trials of SVH responses and for the difference in initial positive displacement estimates of SVV and SVH and in the overall average difference in SVV and SVH estimates. For the SVV estimates, the displacement of the medians (0.5 probability intercepts) in the normal probability plots (Fig. 2A) shows that the SVV estimation was biased in the direction of the initial orientation of the line for that trial. Repeated measures ANOVA and least significant difference (LSD) tests confirmed a preset angle effect; the differences among the overall average, initial positive angle, and initial negative angle displacements were significant (*P* < 0.001). The magnitudes of the biases in final SVV estimates had the same magnitude (but same direction) for an initial positive (CW) or negative (CCW) rotation of the line (Fig. 3, Table 1) and they were within the precision of the adjustment available to the subjects (0.1-degree increments). Hence, the overall average SVV orientation across trials was 0 for the control subjects. This finding suggests that an unbiased estimate of SVV is provided by the overall average of trials with opposite preset angles.

**TABLE 1.** Summary of normative data for average SVV and SVH

Test metric (overall average)	Sex	Mean deviation re: vertical or horizontal, deg	S.D., deg	S.E., deg	Median	1%	2.5%	5%	95%	97.5%	99%
SVV	Both	0	1.722	0.086	0.03	−4.02	−3.52	−2.84	2.53	3.16	4.08
	Female	0.114	1.708	0.139	0.26	−4.47	−3.57	−2.54	2.80	3.21	4.34
	Male	−0.072	1.715	0.11	−0.07	−3.94	−3.66	−3.13	2.53	3.07	5.43
SVH	Both	−0.181	1.642	0.082	−0.19	−4.96	−3.71	−2.95	2.41	2.87	3.50
	Female	−0.185	1.634	0.133	−0.19	−6.38	−4.07	−3.02	2.48	2.82	3.58
	Male	−0.179	1.637	0.105	−0.20	−4.58	−3.70	−2.84	2.32	2.90	3.52
SVV-SVH	Both	0.165	1.121	0.056	0.12	−2.68	−2.09	−1.76	2.14	2.65	3.35
	Female	0.266	1.118	0.091	0.27	−3.08	−1.94	−1.62	2.20	3.27	4.62
	Male	0.107	1.122	0.072	0.06	−2.69	−2.16	−1.85	1.86	2.47	3.04

S.D. indicates standard deviation; S.E., standard error. Percentages are quantiles of the data; SVH, subjective visual horizonal; SVV, subjective visual vertical; SVV-SVN, difference between SVV and SVH.

The SVH estimation was also biased significantly in the direction of the initial line orientation (Fig. 2B), but the magnitudes were smaller than for the SVV (Fig. 3, Table 1). They were also asymmetric, with less bias when the setting was initially positive (CW) by repeated measures ANOVA and LSD tests (*P* < 0.001). The grand mean of individual responses across trials was biased in the negative (CCW) direction.

Deming regression analysis of individual subject responses suggests that the preset bias effect is a static offset of the percept of verticality and horizontality of the initial orientation of the stimulus. Deming regression analysis of SVV with CCW preset as a function of SVV with CW preset was consistent with preset effect, showing an intercept of −0.75 (95% confidence interval [CI], −0.64 to −0.85) and a slope of 0.97 (95% CI, 0.90-1.03), which did not differ significantly from unity. The same analysis for SVH with CCW preset as a function of SVH with CW preset showed a smaller magnitude intercept of −0.40 (95% CI, −0.27 to −0.53) and a slope of 1.01 (95% CI, 0.88-1.04), which also did not differ significantly from unity.

The difference between the SVV and SVH estimates (from vertical or horizontal) gives an estimate of the orthogonality of separate perceptions of up and down. The angles for the trials starting with a positive deviation, trials starting with a negative deviation, and the grand mean of all trials were all significantly different from 0 (1 sample *t* test), with a greater magnitude for trials beginning in the CW (positive) direction. The magnitudes were small, though, and consistent with the bias in the SVV. Deming (linear) regression analyses of estimated SVV as a function of estimated SVH (Table 2) showed a significant offset in the intercept for CW preset estimates of −0.22 degree (95% CI, −0.35 to −0.09) with unity slope, but no corresponding intercept shift for CCW preset trials (0.10 degree, 95% CI, −0.04 to 0.25). Thus, the data suggest that there is a small rotation in perception of SVV (verticality) relative to SVH (horizontality), even in averaged estimates from trials with opposite preset angles.

**TABLE 2.** Regression relationships for subjective visual vertical (SVV) and subjective visual horizonal (SVH)

		Least squares regression			Deming regression	
		Intercept	Slope	R^2^	Intercept (95% CI)	Slope (95% CI)
Average	Both	−0.19	0.77	0.58	*−0.19 (−0.30, −0.08*)	0.94 (0.84, 1.05)
	Female	−0.27	0.78	0.54	−*0.31* (−*0.52,* −*0.10*)^a^	1.08 (0.89, 1.27)
	Male	−0.13	0.72	0.63	−0.12 (−0.25, 0.01)	0.88 (0.76, 1.00)
CW preset	Both	−0.13	0.69	0.53	−*0.22* (−*0.35,* −*0.09*)^a^	0.93 (0.84, 1.03)
	Female	−0.15	0.67	0.46	−*0.31* (−*0.56,* −*0.07*)^a^	0.98 (0.79, 1.16)
	Male	−0.09	0.72	0.59	−0.15 (−0.30, 0.01)	0.92 (0.81, 1.03)
CCW preset	Both	−0.01	0.66	0.44	0.10 (−0.04, 0.25)	0.98 (0.82, 1.14)
	Female	0.04	0.70	0.46	0.15 (−0.10, 0.40)	1.06 (0.88, 1.26)
	Male	−0.04	0.66	0.52	0.05 (−0.13, 0.22)	0.89 (0.72, 1.06)

a *P* < 0.05 from 0.

CI indicates confidence interval.

#### Device-specific Differences

**FIG. 4.**
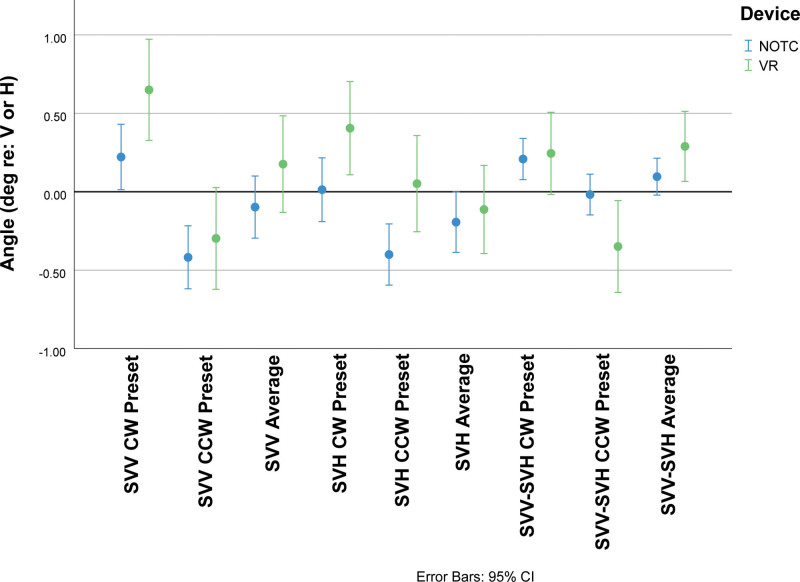
Summary of preset trial responses from the Neuro-otologic Test Center (NOTC) device and the Neurolign Dx 100 (I-PAS) virtual reality (VR) device for subjective visual vertical (SVV) estimates, subjective visual horizontal (SVH) estimates, and the difference between SVV and SVH (SVV-SVH) estimates. The estimates are shown for positive (CW) and negative (CCW) preset values, as well as the average value for SVV and SVH determinations and SVV-SVH values. See text for detailed description. I-PAS indicates I-Portal Portable Assessment System Nystagmograph.

A significant visual preset direction × spatial estimate axis × device interaction effect reflected differences between responses in a real-world estimation device (NOTC device with head-fixed in seat for 258 subjects, 81 females and 177 males) and the head-free VR device (150 subjects, 72 females and 78 males). Group data are shown in Figure 4. Post-hoc LSD tests showed a larger magnitude CW preset bias with the VR device than the NOTC device for both SVV (difference magnitude: 0.43 ± 0.19 [SE] degree, *P* < 0.05) and the SVH (difference magnitude: 0.39 ± 0.18 [SE] degree, *P* < 0.05) and a larger magnitude CCW preset bias for SVH on the NOTC device (difference magnitude: 0.45 ± 0.18 [SE] degree, *P* < 0.05). However, there were no significant NOTC versus VR differences for the averaged SVV or the averaged SVH values (ie, averaged across preset conditions).

#### Sex Differences

**FIG. 5.**
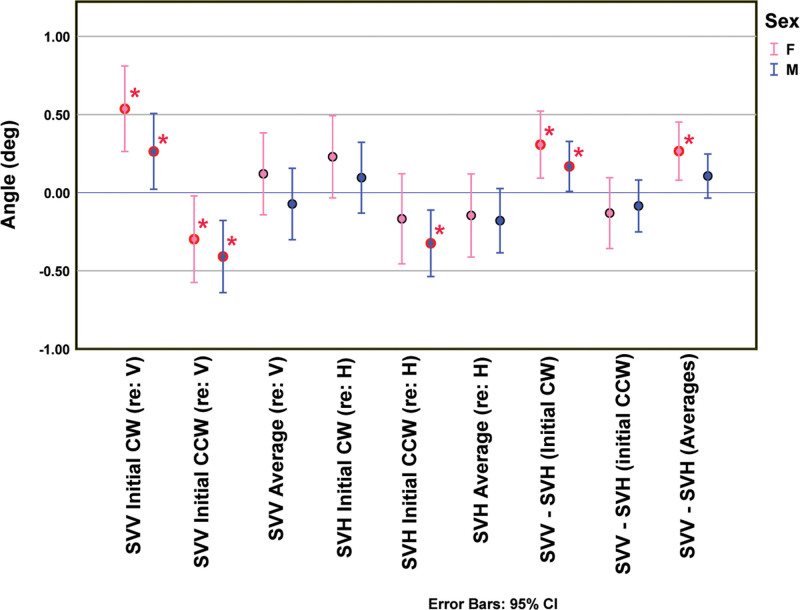
Comparison of subjective visual vertical (SVV) estimates, subjective visual horizontal (SVH) estimates, and the difference between SVV and SVH (SVV-SVH) estimates from female (F) and male (M) subjects. The means and 95% CI of the estimates are shown for positive (CW) and negative (CCW) preset values, as well as the average value for SVV and SVH determinations and SVV-SVH values. Red borders and asterisks indicate significant differences from 0 (*P* < 0.05, 2-sided, t test with false detection rate correction). CI indicates confidence interval.

Kolmogorov-Smirnov tests (Lilliefors correction) retained the null hypothesis of normality for the distributions of the male and female subjects’ SVV responses for initial positive displacement, initial negative displacement, and overall average. For the SVH estimates, the null hypothesis of normality was retained for the female subjects’ estimates of initial positive displacement and overall averages and for the male subjects’ estimates with an initial positive displacement and an initial negative displacement. These tests also retained the hypothesis of normality for the difference in initial positive displacement estimates of SVV and SVH and the difference in initial negative displacement estimates of SVV and SVH among the female subjects and, among the male subjects, in initial positive displacement estimates of SVV and SVH and in the overall average difference SVV and SVH estimates. The group data summary statistics are shown in Table 1 and Figure 5.

ANOVA (repeated measures on estimates, between subjects on sex), followed by LSD comparisons, revealed neither significant main nor interactions effects of subject sex. However, the analyses showed several effects that differed among the trials where the initial line was rotated CW or CCW with respect to the vertical or horizontal orientation. For either female or male subjects, the estimates of either SVV or SVH differed significantly as a function of the initial line orientation (*P* < 0.001). In each case, they were biased in the direction of the initial line orientation (Fig. 5). The SVV estimates deviated significantly from 0 (*P* < 0.05, 2-sided tests, FDR correction) for both sexes and directions, while the SVH differed from 0 only for males with an initial CCW rotation.

There were also significant deviations from orthogonality of SVV and SVH estimates in individual subjects. When the initial rotation was CW, the estimates of SVV differed significantly from estimates of SVH in both females (*P* < 0.005) and males (*P* < 0.05). By contrast, the initial CCW rotation trials showed no significant difference between SVV and SVH estimates in both sexes. Further analysis revealed that the deviation from orthogonality of the SVV and SVH (SVV-SVH) was significantly greater for trials with an initial CW orientation of the test bar than for trials with an initial CCW displacement in both females (*P* < 0.001) and males (*P* < 0.005). These shifts were in the direction of initial bar displacement and were significantly different from 0 for the trials with an initial CW displacement (*P* < 0.05, 2-sided tests, FDR correction) for both females and males. They appear to reflect primarily the magnitude of effects of initial bar orientation on the SVV estimates in both sexes, as opposed to the relative insensitivity of SVH estimates under the same conditions. Finally, the results of Deming regression analyses (of estimated SVV as a function of estimated SVH, Table 2) also show that the initial bar orientation introduced a significant relative offset in the SVV estimate among the female subjects, which was of sufficient magnitude to influence the pooled analysis from males and females.

**FIG. 6.**
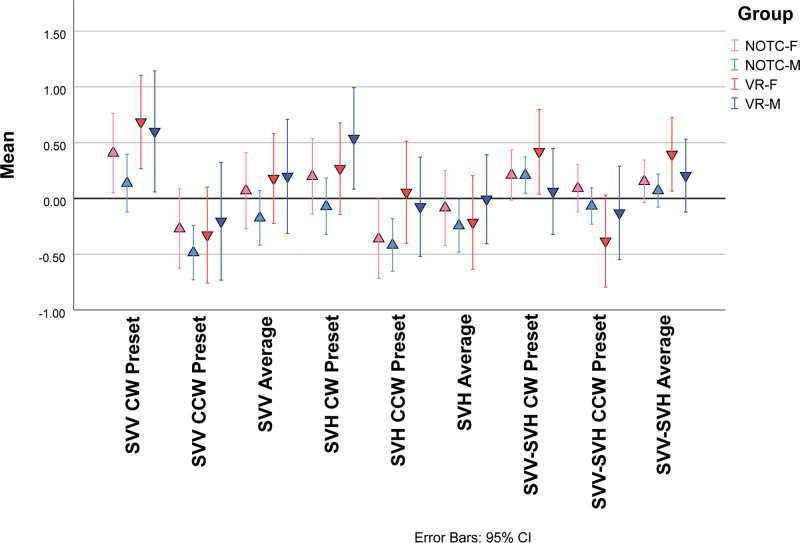
The influences of test device and sex on the preset effect and orthogonality of the SVV and SVH. NOTC-F represents the estimates from females tested with the NOTC device, NOTC-M represents the estimates from males tested with the NOTC device, VR-F represents the estimates from females tested with the Dx-100 device and VR-M represents the estimates from males tested with the Dx 100. See text for a detailed description. NOTC indicates Neuro-otologic Test Center; VR, virtual reality.

Although there were no significant female-male differences in SVV or SVH preset estimates, a visual preset direction × spatial estimate axis × device × sex interaction effect in the overall ANOVA (*F*_1392_ = 7.0, *P* < 0.01) reflected several sex and device-dependent effects of relatively small magnitude (Fig. 6). First, the female subjects showed device-dependent significant differences in the magnitude of preset shifts for the SVV and SVH. For VR presentation only, the SVV in female subjects showed greater magnitude preset effect than the SVH for either CW (difference magnitude: 0.42 ± 0.15 [SE] degree, *P* < 0.01) or CCW (difference magnitude: 0.38 ± 0.16 [SE] degree, *P* < 0.05). In male subjects, by contrast, these effects did not appear. However, for the NOTC presentation only, male subjects showed a small difference in magnitude of SVV versus SVH for the CW present condition (difference magnitude: 0.21 ± 0.10 [SE] degree, *P* < 0.05). There were no sex differences in the estimates of SVV at a given preset direction or SVH at a given preset direction, nor were there significant sex differences in the average SVV or average SVH values with either device.

#### Age-related Differences

For the pooled subjects, there was no correlation between age and CW preset, CCW preset, or average estimate values for either SVV or SVH. Among the male subjects, age was also uncorrelated with the average, CW preset and CCW preset values for either SVV or SVH. Among the female subjects, age was also uncorrelated with the average, CW preset and CCW preset values for SVV and with the average, CW preset values for SVH. However, the CCW preset values for SVH among female subjects showed a small but significant negative correlation (−0.248, *P* < 0.01) with age. Linear regression analysis indicated that *y* intercept was 1.680 ± 0.627 the slope was −0.068 ± 0.022 degree/year. However, the small coefficient of determination (adjusted *R*^2^ = 0.055, *P* > 0.01) indicates that age accounts for only a small percentage of variation SVH estimates in the CCW preset condition in females.

## DISCUSSION

### Overview

This study provides adult normative data (ages 18–50 years old) for identifying clinically abnormal performance on SVV and SVH tests, administered with either a clinical chair system or a VR system. The findings indicate that the average of equal numbers of CW and CCW preset trials was not significantly affected by the test device or sex of the subject for either the SVV or SVH estimates. The preset angle effects for both SVV and SVH and the deviations from orthogonality of SVV and SVH estimates confirmed previous reports. Differences attributable to interactions with device type and sex are of small magnitude and age-related effects were not significant for this population. Because distributional analyses failed to reject the hypothesis of underlying Gaussian distributions for the average SVV and SVH estimates, *z* scores from these distributions can be used to identify clinically normal limits.

### Coordinate Frames for Verticality

Judgments of visual verticality and horizontality are made in the context of the perceived orientation of the head in space. The SVV and SVH tasks were performed on a head-mounted virtual display (Neuro Kinetics I-PAS, Neurolign Dx 100) in the absence of external visuospatial cues about the environment. The subjects were seated and unrestrained, with the head free to move on the trunk and the body free to move in the chair. For these seated subjects, gravitoinertial cues to verticality include pressure on seat and feet, trunk and limb active proprioception relative to gravity, and neck proprioception and jaw muscle proprioception reflecting the mandible as a gravitational load. Because the center of mass of the head is anterior to the atlantooccipital joint and the spinal column, stabilization of the Frankfort plane orientation relative to the spatial horizontal (22–24) necessarily involves neck proprioception, including the added load of the head-mounted display. Hence, the SVV and SVH estimates reflect the situational awareness of the subjects about the orientation of their eyes in space.

### Differences Between Test Devices

The NOTC and virtual display devices differ in several ways that may impact subject responses. For the NOTC device, a subject’s head is fixed with a strap to a headrest while the subject is harnessed to a chair. For the virtual display, the subject is seated without a belt or harness with the head unrestrained. The head-mounted display for this study has integrated pitch, yaw, and roll axis gyroscopes and linear accelerometers to indicate the orientation of the device (and, when worn, the head) in space. The software by default automatically rejects any trial when the head roll is greater than ±2.5 degrees from spatial vertical. These differences in the devices had small but significant influences on the relative magnitude of preset effects in control subjects, consistent with the multisensory nature of these judgments. However, there was no significant effect on the averaged SVV or the averaged SVH values (ie, averaged across preset conditions) that are commonly used for clinical assessment. Hence, a common set of normative value can be used for assessing the averaged SVV and SVH test data across devices. One caveat is that these results were obtained in control subjects. It is not known whether the preset effects may differ in patients with vestibular dysfunction or other neurologic conditions across the different devices.

### Preset Angle Effect

A small, but statistically significant, preset angle effect was confirmed for the SVV for both CW and CCW initial rotations of the light bar. The bias was in the direction of the preset angle and was symmetric (and Gaussian) for pooled male and female subjects (95% CI, CW preset: 0.19-0.55 degree; CCW preset: −0.19 to −0.54 degree). Deming regression estimated individual subject differences in CW versus CCW preset estimates as a linear offset of magnitude of 0.75 degree (95% CI, 0.64-0.85 degree). Further, the average SVV from equal numbers of trials at both presets was consistent with a Gaussian distribution with a mean value of 0 and a standard deviation of 1.72. These estimates are within the ranges from previous recordings of head-upright average SVV (11,15,19,20,25–27). The acceptance of a Gaussian distribution for the overall average SVV value suggests that absolute *z* score (test value/1.72) of magnitude 2 could be used for the detection outliers from normal performance limits at the 95% CI.

The preset effect was less prominent for the SVH estimates and was not symmetric in the different preset directions. For pooled male and female subjects, the overall estimate across preset directions was biased slightly in the CCW direction, at the same magnitude as reported in earlier studies for upright subjects (19,20,28). Across both sexes, the 95% CI for the CW preset SVH trials was −0.03 to 0.31, while the 95% CI for the CCW preset trials was −0.10 to −0.43. The Gaussian hypothesis was accepted for the CW but rejected for the CCW distribution. Deming regression estimated that individual subject differences in the CW versus CCW preset SVH estimates are about half the magnitude of the effect for SVV; the linear offset parameter had a magnitude of 0.40 degree (95% CI, 0.27-0.53 degree). Consistent reported value ranges in the literature (19,20,28) confirm that the SVH is marginally less susceptible to preset bias than the SVV in standard clinical testing conditions. These small biases in SVH estimation are unlikely to affect clinical detection of outliers from the distribution of normal subjects.

The functional significance of a preset effect in subjective determination of verticality and horizontality is not completely understood. For SVV, it is effectively a 0.4-degree underestimate toward the side of the initial visual target tilt relative to gravity. For the SVH task, the underestimate is a bit smaller. From an operational perspective, this slight underestimate appears to be the initial condition for engaging dynamic head movements (in space) to refine the perception of verticality with dynamic haptic, vestibular, and visuospatial cues.

### Orthogonality of SVV and SVH Estimates

The difference from orthogonality between the SVV and SVH estimates was small for either of the preset directions and the averaged values. Deming (linear) regression analyses of estimated SVV as a function of estimated SVH showed only a significant offset effect for the CW preset condition. This replicates other findings in the literature for upright subjects (20) and suggests that approximate orthogonality of the SVV and SVH estimates are expected for normal subjects in the clinical test environment. Further, a Kolmogorov-Smirnov test retained the hypothesis of a Gaussian distribution for the deviation from orthogonality of the overall SVV and the overall SVH estimates for each subject. Therefore, an absolute *z* score for the deviation from orthogonality of magnitude ±2 (equivalent to less than –2.26 degrees or greater than 2.59 degrees [CW positive convention]) is a criterion for detection of an outside normal limits discrepancy between SVV and SVH determinations by an individual.

### Sex-associated Effects

The differences underlying the identified sex-associated interaction effects are empirical findings of relatively low magnitude and are worthy of exploration in association with body tilt and larger numbers of preset trials. There is a clinically relevant implication, though. Because there were no significant sex differences in either the averaged SVV estimates or the averaged SVH estimates, the pooled findings from female and male subjects are appropriate for clinical determinations of the averaged SVV and averaged SVH performances relative to normal limits from the parameters of the underlying Gaussian distributions.

### Age-associated Effects

There is no significant age effect for both males and females for the average CW preset and CCW preset values for SVV or SVH. For males, there was no difference in the average value for the CCW preset. However, there was a small but significant negative correlation of these values among female subjects. Despite this one significant effect, it must be pointed out that this age-related difference only accounts for a very small parentage of the variability of outcomes and only for this 1 measurement in female individuals. This finding implies that normative values do not have to be tightly controlled within age cohorts for adults below the age of 50 years for setting boundaries for normal or abnormal performance.

### Limitations

The study is a post-hoc analysis of control data pooled from multiple centers and studies. Hence, the cross-sectional findings are limited to the age range of this group, adults between the ages of 18–50 years. Given the prevalence of balance dysfunction with aging, there is a need for a large-sample normative study of SVV and SVH estimated in geriatric patients (with strict inclusion criteria). The design of such a study could include factors such as non-otologic disease burden. These normative measures are also limited to the protocols and test devices for the determinations. The patients at different sites and from different control study cohorts were exposed to the same test protocol (as part of different test suites on different platforms) and show the robustness of the measures under those conditions. Within these limits, the normative values serve as a guide for objective identification of performance outside normal limits and for exploration of the potential differential diagnostic utility of preset effects in clinical trajectories of acute, subacute, and chronic patient populations.

## CONCLUSIONS

Judgments of the SVV and SVH are standard components of clinical test batteries for vestibular function. These tests are conducted on an upright patient in a seated position. In the current study, we used data from clinically healthy males and females, aged 18–50 years, to assess the statistical distribution of data from these tests and the magnitudes of the preset effect, sex differences, and fixed-head versus head-free device platforms in these determinations. Our findings indicated no age-related effects on SVV and SVH in this population. The preset angle effects noted for both SVV and SVH and their deviations from orthogonality agree in magnitude with previous research. Differences associated with device type, age, and sex, though statistically significant, were of small magnitude in the stationary, upright subjects, suggesting that these variables have limited influence on the SVV and SVH clinical findings. The findings also confirmed that the average of equal numbers of CW and CCW preset trials, a common clinical measure for SVV and SVH, was not significantly affected by the test device, age, or sex of the subject. Finally, the acceptance of underlying Gaussian distributions of the SVV and SVH measures establishes that *z* scores can be used by clinicians to objectively detect patients who may be outliers from normal functional limits. It is noteworthy that the recommended range for identifying normal SVV or SVH performance with these devices (±3 degrees) is highly consistent with the quantile distribution of the normative data in Table 1. Other cutoffs for abnormal SVH in published studies, such as ±2.5 degrees (29), simply reflect a different likelihood of false negative findings.

The robust statistical norms for the SVV and SVH in adults (18–50 years old) anchor their role as a basic psychophysical screening test in assessment of patients with suspected vestibular dysfunction. The principles underlying clinical interpretation of the SVV in diagnosis of peripheral and central vestibular dysfunction were reviewed rigorously by Dieterich and Brandt (18). The summary of their review of pathological SVV findings in Table 1 (18) gives a perspective for the direct application of our statistical findings to clinical practice. First, the reported SVV means/medians for patients with central or peripheral disorders exceeded the 95% CI for the control patients in our study. Second, the reported ranges of SVV findings for cerebral cortical, thalamic midbrain, pontomesencephalic, pontine, medullary, brainstem disorder patients or labyrinth/nerve disorder patients showed only minimal overlap with the 95th percentile of normative data. This minimal overlap could be either statistical outliers (30) from a clinical (abnormal) population or false negative findings by the test. The current data identify the false positive likelihood of an abnormal score from a test session from a *z* score of the estimated Gaussian distribution. It is left to the physician to select an acceptable probability level for designating the test as abnormal.

## FUNDING SOURCES

None declared.

## CONFLICT OF INTEREST STATEMENT

Alexander Kiderman is Chief Technology Officer for, and shareholder of Neurolign USA. His contributions were limited to device design and technical aspects of manuscript preparation. No conflicts are declared by the other authors.

## DATA AVAILABILITY STATEMENT

The datasets generated during and/or analyzed during the current study are not publicly available, but are available from the corresponding author on reasonable request.
